# Evidence for an ependymoma tumour suppressor gene in chromosome region 22pter–22q11.2

**DOI:** 10.1038/sj.bjc.6690822

**Published:** 1999-12

**Authors:** T J M Hulsebos, N T Oskam, E H Bijleveld, A Westerveld, M A Hermsen, A M W van den Ouweland, B C Hamel

**Affiliations:** Department of Human Genetics, University of Amsterdam, Meibergdreef 15, 1105 AZ Amsterdam, The Netherlands; Department of Pathology, Free University, Amsterdam, The Netherlands; Department of Clinical Genetics, Erasmus University, Rotterdam, The Netherlands; Department of Human Genetics, University Hospital, Nijmegen, The Netherlands; Department of Neurology, St Elisabeth Hospital, Tilburg, The Netherlands

**Keywords:** ependymoma, familial clustering, tumour suppressor gene, chromosome 22

## Abstract

Ependymomas are glial tumours of the brain and spinal cord. The most frequent genetic change in sporadic ependymoma is monosomy 22, suggesting the presence of an ependymoma tumour suppressor gene on that chromosome. Clustering of ependymomas has been reported to occur in some families. From an earlier study in a family in which four cousins developed an ependymoma, we concluded that an ependymoma-susceptibility gene, which is not the *NF2* gene in 22q12, might be located on chromosome 22. To localize that gene, we performed a segregation analysis with chromosome 22 markers in this family. This analysis revealed that the susceptibility gene may be located proximal to marker *D22S941* in 22pter–22q11.2. Comparative genomic hybridization showed that monosomy 22 was the sole detectable genetic aberration in the tumour of one of the patients. Loss of heterozygosity studies in that tumour revealed that, in accordance to Knudson’s two-hit theory of tumorigenesis, the lost chromosome 22 originated from the parent presumed to have contributed the wild-type allele of the susceptibility gene. Thus, our segregation and tumour studies collectively indicate that an ependymoma tumour suppressor gene may be present in region 22pter–22q11.2. © 1999 Cancer Research Campaign


*British Journal of Cancer* (1999) **81**(7), 1150–1154
© 1999 Cancer Research Campaign
Article no. bjoc.1999.0822

### Evidence for an ependymoma tumour suppressor gene in chromosome region 22pter–22q11.2

**TJM Hulsebos[1], NT Oskam[1], EH Bijleveld[1], A Westerveld[1], MA Hermsen[2], AMW van den Ouweland[3], BC Hamel[4] and CC Tijssen[5]**

[1]Department of Human Genetics, University of Amsterdam, Meibergdreef 15, 1105 AZ Amsterdam, The Netherlands; [2]Department of Pathology, Free University, Amsterdam, The Netherlands; [3]Department of Clinical Genetics, Erasmus University, Rotterdam, The Netherlands; [4]Department of Human Genetics, University Hospital, Nijmegen, The Netherlands; [5]Department of Neurology, St Elisabeth Hospital, Tilburg, The Netherlands

**Summary** Ependymomas are glial tumours of the brain and spinal cord. The most frequent genetic change in sporadic ependymoma is monosomy 22, suggesting the presence of an ependymoma tumour suppressor gene on that chromosome. Clustering of ependymomas has been reported to occur in some families. From an earlier study in a family in which four cousins developed an ependymoma, we concluded that an ependymoma-susceptibility gene, which is not the *NF2* gene in 22q12, might be located on chromosome 22. To localize that gene, we performed a segregation analysis with chromosome 22 markers in this family. This analysis revealed that the susceptibility gene may be located proximal to marker *D22S941* in 22pter–22q11.2. Comparative genomic hybridization showed that monosomy 22 was the sole detectable genetic aberration in the tumour of one of the patients. Loss of heterozygosity studies in that tumour revealed that, in accordance to Knudson's two-hit theory of tumorigenesis, the lost chromosome 22 originated from the parent presumed to have contributed the wild-type allele of the susceptibility gene. Thus, our segregation and tumour studies collectively indicate that an ependymoma tumour suppressor gene may be present in region 22pter–22q11.2. © 1999 Cancer Research Campaign

**Keywords:** ependymoma; familial clustering; tumour suppressor gene; chromosome 22

Ependymomas are glial neoplasms developing from ependymal cells that line the cerebral ventricles and the central canal of the spinal cord. They are most commonly seen in children and adolescents in which they account for about 10% of all brain tumours (Russell and Rubinstein, 1989). Only a limited number of genetic changes have been reported for this type of tumour. Loss of heterozygosity for chromosome 22 (LOH 22) is a rather frequent event (about 30% of cases) in adult ependymomas but is infrequent in pediatric ependymomas (Bijlsma et al, 1995; Von Haken et al, 1996). Neurofibromatosis type 2 (NF2) patients are at increased risk of developing a spinal ependymoma (Martuza et al, 1988). Indeed, mutations in the *NF2* gene on chromosome 22 have been found in sporadic spinal cord ependymomas, but not in sporadic intracranial ependymomas (Rubio et al, 1994; Ng et al, 1995; Slavc et al, 1995; Birch et al, 1996; Von Haken et al, 1996). *TP53* gene mutations are rare in sporadic intracranial as well as spinal ependymomas (Von Haken et al, 1996; Nozaki et al, 1998). In one study, frequent LOH was found for the region on chromosome arm 17p distal to the *TP53* gene (Von Haken et al, 1996). No other regions or genes have been identified as being frequently involved in ependymoma tumorigenesis (Bijlsma et al, 1995; for review, see Hamilton and Pollack, 1997).

A consistent cytogenetic aberration in a tumour may point to the presence of a gene that is involved in the generation of that tumour. In this respect, Weremowicz et al (1992) found in the anaplastic ependymoma of a 5-year-old boy loss of one copy of chromosome 22 and a balanced 1/22 translocation with the breakpoint at 22q13.3 in the retained homologue. This suggests the presence of an ependymoma tumour suppressor gene in the telomeric part of chromosome 22. On the other hand, a patient with a malignant ependymoma at age 5 has been reported to carry a balanced constitutional t(1;22) (p22; q11.2) with the chromosome 22 breakpoint between *ARVCF* and *D22S264* in the centromeric part of chromosome arm 22q (Park et al, 1996; Rhodes et al, 1997). This may indicate that an ependymoma-causative gene is located at that position.

Clustering of ependymomas has been noted in some families, suggesting inheritance of a genetic susceptibility to this type of tumour. Honan et al (1987) reported on a family of 11 siblings, of when five developed brain tumours: three had a subependymoma, one an ependymoma and one a brain tumour of unknown pathology. In another family, two sisters and their mother's male cousin all developed an ependymoma (Savard and Gilchrist, 1989). One of the sisters had monosomy 22 in the tumour. Ryken et al (1994) presented in their study a father and son, who both developed a subependymoma. Finally, we reported previously on a family with four male cousins, whose fathers were brothers, with verified anaplastic ependymoma in three cases and evidence of ependymoma in the fourth case (Nijssen et al, 1994). Monosomy 22, but no mutation in the *NF2* gene, was found in the ependymoma of one of the patients.

Taken together, the available data suggest that an ependymma-related tumour suppressor gene, of which the mutated form segregates in ependymoma families and which is inactivated by loss in the tumour, might be present on chromosome 22. To localize that gene, we performed a segregation analysis, using a set of markers that covers the whole chromosome 22, in the ependymoma family that was presented previously (Nijssen et al, 1994).

*Received 12 January 1999*
*Revised 19 April 1999*
*Accepted 22 April 1999*

*Correspondence to:* TJM Hulsebos

In addition, we analysed the ependymomas of two patients in this family for loss of chromosome 22 markers. From these analyses we conclude that an ependymoma tumour suppressor gene may be present in region 22pter-22q11.2.

### MATERIALS AND METHODS

### Family study and clinical data

The pedigree of the family is shown in Figure 1. The clinical data for the family members have been presented previously (Nijssen et al, 1994). In short, no signs of neurofibromatosis were present in this family. Individuals III-1, III-3 and III-4 developed a histopathologically proven intracranial anaplastic ependymoma at young age (< 5 years). Computerized tomography (CT) scanning showed a large tumour in the fourth ventricle of patient III-2 at 6 months of age, which was not evident at 3 days after birth. Histopathological data were not available for this tumour. CT and magnetic resonance imaging (MRI) showed in patient III-4 at the age of 8 months an infratentorial tumour, although cerebral CT was normal at the age of 2 months. Since the earlier CT scans of two patients showed no significant abnormalities, we think that the ependymomas were anaplastic from the beginning. Cerebral MRI of individual II-3 was normal.

### Molecular genetics and cytogenetics

Genomic DNA was extracted from blood leucocytes of the family members and the frozen tumour of patient III-4 as described previously (Bijlsma et al, 1995). For patient III-1, only formalin-fixed and paraffin-embedded tumour material was available from which genomic DNA was isolated according to Speicher et al (1995). Tumour and normal tissue of patient III-3 was not available. Comparative genomic hybridization (CGH) for the tumour of patient III-4 was performed as described (Hermsen et al, 1997). For microsatellite analysis, primer sequences and polymerase chain reaction (PCR) conditions were taken from the relevant databases by accession through the Internet. The order of markers on chromosome 22 used in this study was deduced from published maps (Collins et al, 1995; Dib et al, 1996). The known cytogenetic localizations of markers were taken from the Genome Database and are shown in Figure 2. PCR was performed essentially as described in Bijlsma et al (1995). LOH in the tumours was determined by visual inspection of autoradiographs. Due to considerable degradation of the DNA extracted from the tumour of patient III-1, only PCRs generating products smaller than about 150 bp could be executed using this DNA as template.

### RESULTS

Interphase fluorescence in situ hybridization analysis on selected chromosomes in the tumour of patient III-4 suggested in our previous study monosomy 22 and normal copy numbers for chromosomes 1, 6, 7, 10, 11, 15, 17, 18, X and Y (Nijssen et al, 1994). To confirm and extend these observations, we performed CGH analysis of this tumour. The CGH profile for each chromosome is shown in Figure 3. Apart from monosomy 22, no other tumour-related chromosomal changes are present, suggesting that loss of one copy of chromosome 22 is the sole genetic aberration in this tumour.

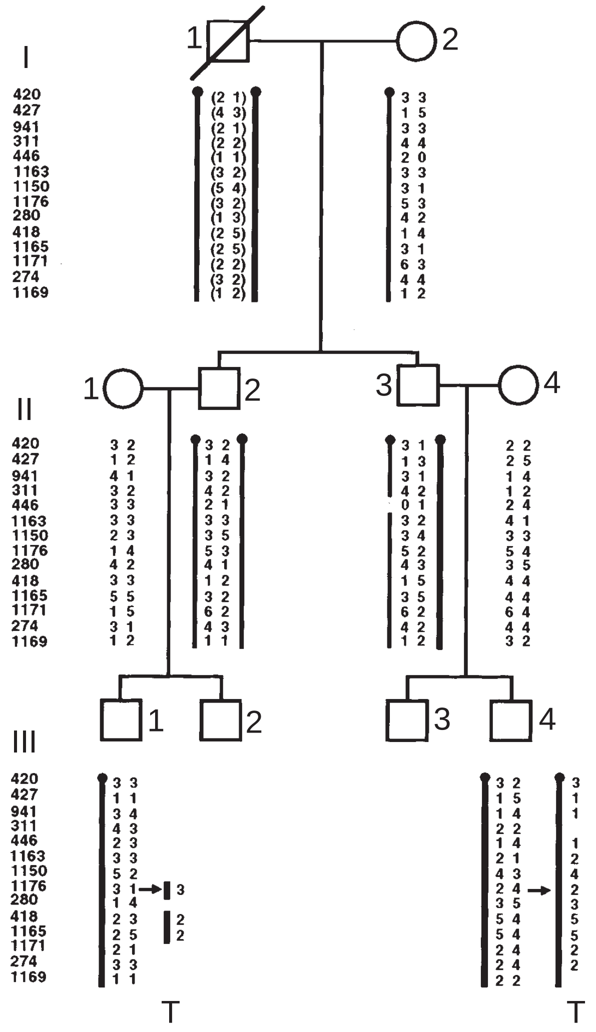

**Figure 1** Genotype analysis of the family using markers at loci on chromosome 22. T denotes marker alleles retained in the tumour. Grey symbols represent patients with ependymoma. To follow their segregation in the family, the grandparental chromosomes are indicated by differently coloured bars. Haplotypes for each chromosome were constructed by assuming minimal numbers of recombinations. The most probable haplotypes for the grandpaternal chromosomes were inferred from those of children and spouse. Genotypes for marker *D22S446* suggest absence of one allele (indicated by 0) in the constitutional DNA of individuals I-2 and II-3. Since individual I-2 is homozygous for *D22S941*, *D22S311*, and *D22S1163* the recombination events, evident in the constitutional DNA of II-3, could also have taken place in the region defined by these markers. The segment that is shared by patients III-1 and III-4 is limited to the region defined by markers *D22S420* and *D22S427* in the centromeric part of chromosome arm 22q

According to Knudson's two-hit model of tumorigenesis, as adapted by Cavenee et al (1983), loss of chromosome 22 represents the second hit, i.e. loss of the wild-type allele of the presumed tumour suppressor gene. To map the first hit, i.e. the inactivated allele that segregates in the family, we performed a genetic analysis of the family members using microsatellite markers at loci that cover the whole chromosome 22. The results are summarized in Figure 1. As an example, the genotyping of the family members for marker *D22S427* is shown in Figure 4A.

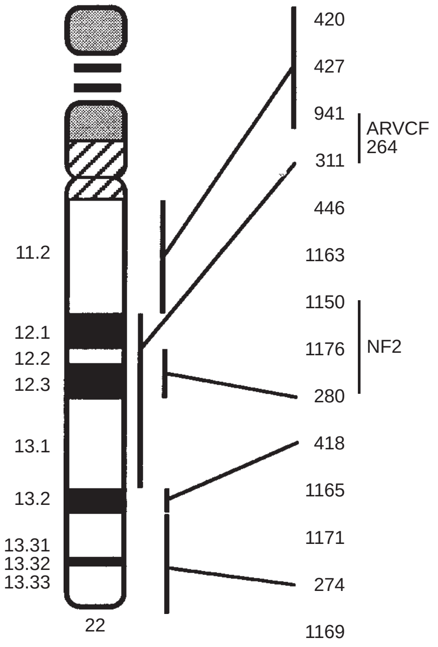

**Figure 2** Linear order and cytogenetic localization of the chromosome 22 markers used in this study. The positions of *ARVCF*, *D22S264*, and *NF2*, which are discussed in the text, are indicated

Haplotypes for the various chromosomes 22 were constructed by assuming minimal numbers of recombinations. The two affected individuals III-1 and III-4 only share the segment of chromosome 22 that is defined by alleles 3 and 1 of markers *D22S420* and *D22S427* respectively. These alleles originate from their grandmother (I-2).

We analysed the tumours of patients III-1 and III-4 for LOH of the chromosome 22 markers that were used in the segregation analysis. Every informative marker displayed LOH in the tumour of patient III-4 and the lost allele was for each marker of maternal origin (Figures 1 and 4). Unfortunately, because of heavily degraded DNA, the LOH status of only three markers could be successfully determined in the tumour of patient III-1. All three markers displayed LOH in the tumour, suggesting that at least the central and telomeric part of chromosome 22 are also lost in that tumour. The lost alleles originate from the mother of the patient. As shown in Figure 4B, the retained allele of marker *D22S1176*, which is in the *NF2* gene region in 22q12 (Figure 2), is different in the two tumours and both alleles originate from the grandfather (I-1).

### DISCUSSION

If one assumes that in the family studied here the histopathologically verified presence of an ependymoma in three of the four cousins is not merely a coincidence but the consequence of the inheritance of a mutated susceptibility gene for that tumour, then the region 22pter-22q11.2 proximal to *D22S941* and including

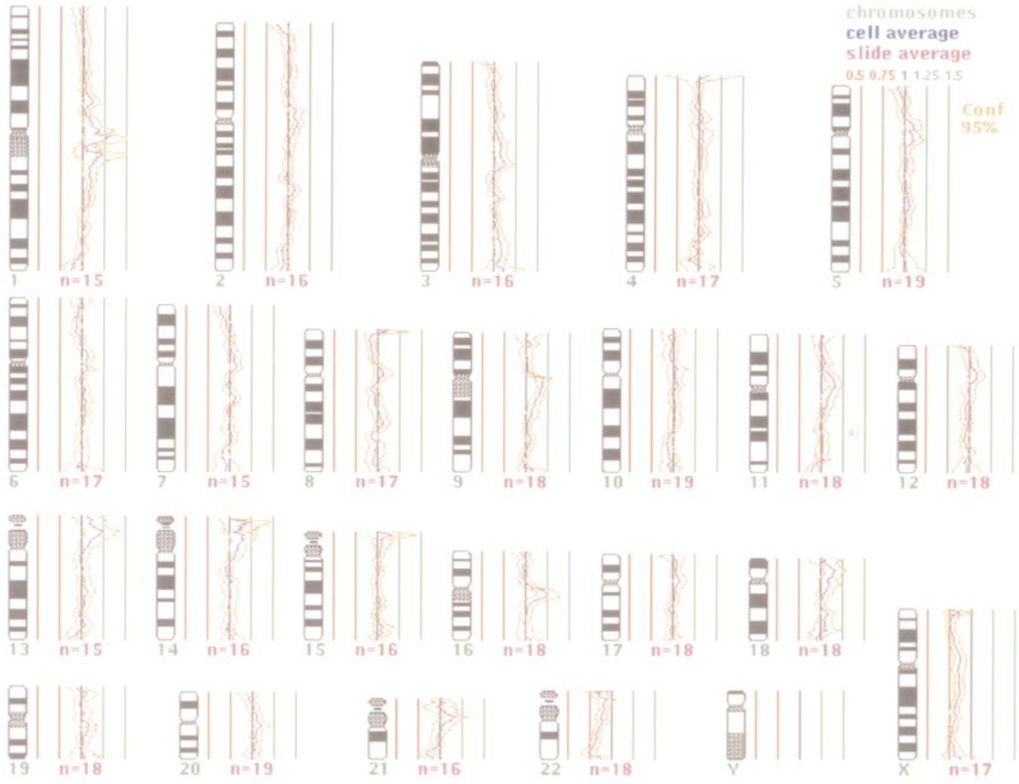

**Figure 3** CGH analysis of the tumour of patient III-4. The highly polymorphic centromeric and heterochromatic regions are excluded from CGH analysis because of technical reasons. The figure beneath each profile indicates number of chromosomes analysed to obtain the average profile shown. Normal female DNA was used as reference DNA. Since the tumour DNA was from a male patient, the profile for the X-chromosome shows apparent loss of one copy in the tumour. Apart from this, the sole aberration in the tumour is loss of one copy of chromosome 22

 *© 1999 Cancer Research Campaign*

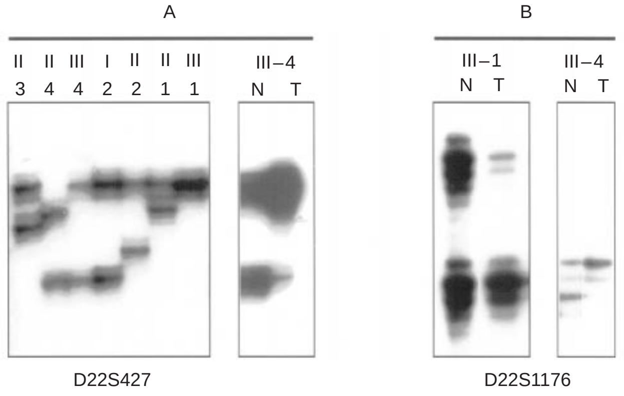

**Figure 4** Autoradiographs showing the genotype analysis of family members and the LOH analysis of the tumour of patient III-4 for marker *D22S427* (**A**) and the LOH analysis of the tumours of patients III-1 and III-4 for marker *D22S1176* (**B**). N, normal DNA; T, tumour DNA. Deduced genotypes are shown in Figure 1. LOH analyses display loss of allele 5 of marker *D22S427* in the tumour of patient III-4 and loss of alleles 1 and 4 of marker *D22S1176* in the tumour of patients III-1 and III-4 respectively.

*D22S420* and *D22S427* is the most likely location of that gene. This segment is shared by patients III-1 and III-4, and is inherited from their grandmother. All other chromosome 22 segments in the constitutional DNA of these patients are from different chromosomes, thereby excluding one origin. In accordance with the tumour suppressor gene model, the maternal chromosome 22 copy, carrying the wild-type allele, is lost and the grandmaternal chromosome 22 segment, with the presumed inactivated allele, is retained in the tumour of patient III-4 (Figure 1). This is exemplified in Figure 4A by loss in the tumour of the maternal allele 5 of marker *D22S427* in the commonly inherited region. In addition, in case of marker *D22S1176*, different grandpaternal alleles are retained in the tumours of patients III-1 and III-4, strongly suggesting that the closely linked *NF2* gene copies also are of different origin (Figure 4B). This excludes the *NF2* gene as the common susceptibility factor, which is in accordance with our earlier failure to find a mutation in the *NF2* gene in the tumour of patient III-4 (Nijssen et al, 1994).

The most probable location of the ependymoma-susceptibility gene is proximal to *D22S941* (Figure 1). The chromosome 22 breakpoint of the constitutional t(1;22) (p22;q11.2) in the young patient with a malignant ependymoma has been mapped between *ARVCF* and *D22S264* (Rhodes et al, 1997). Considering the order cen — *D22S941* — *ARVCF* — *D22S264* — *D22S311* — tel (Sirotkin et al, 1997), this would indicate that two different genes are implicated in ependymoma tumorigenesis. Since the precise physical distances in the region under study are not known yet, the presence of a very large gene that is inactivated by mutation in our family and by translocation in the sporadic tumour cannot be excluded at the moment. Alternatively, as suggested by Rhodes et al (1997), it is possible that the translocation in their patient causes the activation of the oncogene, rather than the inactivation of a tumour suppressor gene, which might be located on chromosome 1 and not on chromosome 22.

It is clear from the absence of affected individuals in generations I and II that the development of ependymomas in generation III in the studied family cannot simply be explained by the mendelian inheritance of a single ependymoma-susceptibility gene. Absence of disease in earlier generations has also been documented for other (sub)ependymoma families (Honan et al, 1987; Savard and Gilchrist, 1989). These family data suggest multifactorial inheritance in which ependymoma development is determined by two or more loci. The one on chromosome 22 contributes significantly to the disease phenotype and both copies of that gene must be inactivated for tumour formation to occur.

The distal boundary of the ependymoma-susceptibility gene region is defined by the recombination event between *D22S427* and *D22S941* in individual II-3, as detected in the constitutional DNA of III-4 (Figure 1). Both markers are within the centromeric region of chromosome arm 22q that includes the Cat Eye and DiGeorge Syndrome Critical Regions. The complete nucleotide sequence of the latter regions is currently being determined (B Roe et al, personal communication). This sequencing effort will certainly reveal many new candidate genes, which will be tested for their involvement in ependymoma tumorigenesis.

### ACKNOWLEDGEMENTS

We thank Dr JLJM Teepen (Department of Pathology, St Elisabeth Hospital, Tilburg) and Dr E Zwarthoff (Department of Pathology, Erasmus University, Rotterdam) for providing us with tumour material of patients III-1 and III-4 respectively. We gratefully acknowledge the expert technical assistance of M van Dartel and S Redeker. This work was supported by grant AMC 94-700 from the Dutch Cancer Society.

### REFERENCES

Birch BD, Johnson JP, Parsa A, Desai RD, Yoon JT, Lycette CA, Li YM and Bruce JN (1996) Frequent type 2 neurofibromatosis gene transcript mutations in sporadic intramedullary spinal cord ependymomas. *Neurosurgery* **39**: 135–140

Bijlsma EK, Voesten AMJ, Bijleveld EH, Troost D, Westerveld A, Mérel P, Thomas G and Hulsebos TJM (1995) Molecular analysis of genetic changes in ependymomas. *Genes Chromosomes Cancer* **13**: 272–277

Cavenee WK, Dryja TP, Phillips RA, Benedict WF, Godbout R, Gallie BL, Murphree AL, Strong LC and White RL (1983) Expression of recessive alleles by chromosomal mechanisms in retinoblastoma. *Nature* **305**: 779–784

Collins JE, Cole CG, Smink LJ, Garrett CL, Leversha MA, Soderlund CA, Masien GL, Everett LA, Rice KM, Coffey AJ, Gregory SG, Gwilliam R, Dunham A, Davies AF, Hassock S, Todd CM, Lehrach H, Hulsebos TJM, Weissenbach J, Morrow B, Kucherlapati RS, Wadey R, Scambler PJ, Kim U-J, Simon MI, Peyrard M, Xie Y-G, Carter NP, Durbin R, Dumanski JP, Bentley DR and Dunham I (1995) A high-density YAC contig map of human chromosome 22. *Nature* **377**: 367S–379S

Dib C, Faure S, Fizames C, Samson D, Drouot N, Vignal A, Millasseau P, Marc S, Hazan J, Seboun E, Lathrop M, Gyapay G, Morissette J and Weissenbach J (1996) A comprehensive genetic map of the human genome based on 5,264 microsatellites. *Nature* **380**: 152–154

Hamilton RL and Pollack IF (1997) The molecular biology of ependymomas. *Brain Pathol* **7**: 807–822

Hermsen MA, Joenje H, Arwert F, Braakhuis BJ, Baak JP, Westerveld A and Slater R (1997) Assessment of chromosomal gains and losses in oral squamous cell carcinoma by comparative genomic hybridisation. *Oral Oncol* **33**: 414–418

Honan WP, Anderson M, Carey MP and Williams B (1987) Familial subependymomas. *Br J Neurosurg* **1**: 317–321

Martuza RL and Eldridge R (1988) Neurofibromatosis 2 (Bilateral acoustic neurofibromatosis). *N Engl J Med* **318**: 684–688

Ng H, Lau K, Tse JYM, Lo K, Wong JHC, Poon W and Huang DP (1995) Combined molecular genetic studies of chromosome 22q and the neurofibromatosis type 2 gene in central nervous system tumors. *Neurosurgery* **37**: 764–773

Nozaki M, Tada M, Matsumoto R, Sawamura Y, Abe H and Iggo RD (1998) Rare occurrence of inactivating p53 gene mutations in primary non-astrocytic tumors of the central nervous system: reappraisal by yeast functional assay. *Acta Neuropathol* **95**: 291–296

© 1999 Cancer Research Campaign

Nijssen PCG, Lekanne Deprez RH, Tijssen CC, Hagemeijer A, Arnoldus EPJ, Teepen JLJM, Holl R and Niermeyer MF (1994) Familial anaplastic ependymoma: evidence of loss of chromosome 22 in tumour cells. *J Neurol Neusosurg Psychiatry* **57**: 1245–1248

Park JP, Chaffee S, Noll WW and Rhodes CH (1996) Constitutional de novo t (1;22) (p22; q11.2) and ependymoma. *Cancer Genet Cytogenet* **86**: 150–152

Rhodes CH, Call KM, Budarf ML, Barnoski BL, Bell CJ, Emanuel BS, Bigner SH, Park JP and Mohandas TK (1997) Molecular studies of an ependymoma-associated constitutional t (1; 22) (p22; q11.2). *Cytogenet Cell Genet* **78**: 247–252

Rubio M-P, Correa KM, Ramesh V, MacCollin MM, Jacoby LB, Von Deimling A, Gusella JF and Louis DN (1994) Analysis of the neurofibromatosis 2 gene in human ependymomas and astrocytomas. *Cancer Res* **54**: 45–47

Russell DS and Rubinstein LJ (1989) Tumours of the ependymoma group. In: *Pathology of Tumours of the Nervous System*, 5th edn, pp. 187–219. Edward Arnold: London

Ryken TC, Robinson RA and VanGilder JC (1994) Familial occurrence of subependymoma. Report of two cases. *J Neurosurg* **80**: 1108–1111

Savard ML and Gilchrist DM (1989) Ependymomas in two sisters and a maternal male cousin with mosaicism with monosomy 22 in tumour. *Pediatr Neurosci* **15**: 80–84

Sirotkin H, O'Donnell H, DasGupta R, Halford S, St. Jore B, Puech A, Parimoo S, Morrow B, Skoultchi A, Weissman SM, Scambler P and Kucherlapati R (1997) Identification of a new catenin gene family member (ARVCF) from the region deleted in Velo-Cardio-Facial Syndrome. *Genomics* **41**: 75–83

Slavc I, MacCollin MM, Dunn M, Jones S, Sutton L, Gusella JF and Biegel JA (1995) Exon scanning for mutations of the NF2 gene in pediatric ependymomas, rhabdoid tumors and meningiomas. *Int J Cancer* **64**: 243–247

Speicher MR, Jauch A, Walt H, du Manoir S, Ried T, Jochum W, Sulser T and Cremer T (1995) Correlation of microscopic phenotype with genotype in a formalin-fixed, paraffin-embedded testicular germ cell tumor with universal DNA amplification, comparative genomic hybridization, and interphase cytogenetics. *Am J Pathol* **146**: 1332–1340

Von Haken MS, White EC, Daneshvar-Shyesther L, Sih S, Choi E, Kalra R and Cogen PH (1996) Molecular genetic analysis of chromosome arm 17p and chromosome arm 22q sequences in sporadic pediatric ependymomas *Genes Chromosomes Cancer* **17**: 37–44

Weremowicz S, Kupsky WJ, Morton CC and Fletcher JA (1992) Cytogenetic evidence for a chromosome 22 tumor suppressor gene in ependymoma. *Cancer Genet Cytogenet* **61**: 193–196

 *© 1999 Cancer Research Campaign*
